# Beta-glucan promotes dental pulp healing by enhancing cell proliferation, migration, and mineralization

**DOI:** 10.1016/j.jds.2024.12.007

**Published:** 2024-12-16

**Authors:** Taweesak Kaokai, Jitjiroj Ittichaicharoen, Tanida Srisuwan

**Affiliations:** a Dental Department, Phobphra Hospital, Tak, Thailand; b Department of Oral Medicine and Diagnostic Science, Chiang Mai University, Chiang Mai, Thailand; c Department of Restorative Dentistry and Periodontology, Chiang Mai University, Chiang Mai, Thailand

**Keywords:** Beta-glucan, Fibroblast, Human dental pulp cell, Vital pulp therapy, Wound healing

## Abstract

**Background/purpose:**

Effective dental pulp healing is essential for preserving tooth vitality. Although beta-glucan has shown promise in wound healing in the medical fields, its potential effects on human dental pulp cells (HDPCs) remain unexplored. This study aimed to assess beta-glucan's effects on HDPC proliferation, migration, collagen synthesis, mineralization, and differentiation.

**Materials and methods:**

Primary HDPCs were cultured and assigned into five groups: control, vehicle, and beta-glucan at concentrations of 5, 7.5, and 10 mg/mL. Cell proliferation was quantified using the alamarBlue® assay at 24, 48, and 72 h. Cell migration was assessed at 12 and 24 h via the scratch wound healing assay. Flow cytometry was employed to detect integrin beta 1 (CD29) expression during wound healing. Mineralization and differentiation at day 14 were evaluated through alizarin red S staining and quantitative real-time polymerase chain reaction (qRT-PCR), measuring Dentin Sialophosphoprotein (DSPP), Interleukin-10 (IL-10), and Collagen type I (COL1) gene expression. Statistical significance was established at *P* < 0.05.

**Results:**

At 24 and 72 h, all concentrations of beta-glucan significantly induced cell proliferation. In the wound healing assay, beta-glucan improved cell migration and increased the expression of integrin beta 1 after 24 h. Mineralized matrix formation and the expression of IL-10 and COL1 were significantly observed at 14 days. The upregulation of DSPP was detected in groups supplemented with 5 and 7.5 mg/mL beta-glucan.

**Conclusion:**

Beta-glucan enhanced cell proliferation, cell migration potential, integrin beta 1 expression, mineralized matrix formation, and DSPP, IL-10, and COL1 gene expression in HDPCs.

## Introduction

Vital pulp therapy (VPT) aims to maintain the vitality of teeth affected by deep caries, trauma, or iatrogenic errors.^1^ Preserving pulp vitality is beneficial as it provides a defense against external harms and supports the self-protective and reparative processes when injured.^2^^,^^3^ Modern VPT involves removing infected pulp tissue and applying a bioactive capping material to the exposed pulp.^4^, ^5^, ^6^ The potential for pulpal wound healing is crucial post-VPT due to the uncertain condition of the pulp after infected tissue removal. Dental pulp stem cells at the injury site are believed to contribute to the initial repair process.^7^^,^^8^ However, information on the reparative potential of pulp tissue healing, particularly wound closure after VPT, is limited. Therefore, supplements, techniques, and innovations that promote initial wound healing could significantly benefit VPT.

Beta-glucans, natural polysaccharides found in plants, bacteria, fungi, and algae, have gained attention for their therapeutic potential in wound healing.^9^ These compounds exhibit remarkable biological properties, including the ability to enhance critical cellular processes such as cell proliferation, migration, reepithelization, angiogenesis, and collagen synthesis.^10^, ^11^, ^12^ As a result, beta-glucans are emerging as promising candidates for the development of natural wound-healing agents in the medical field. Clinical studies have shown that beta-glucan applications can accelerate wound closure and reduce treatment costs, particularly for chronic wounds.^13^^,^^14^ Additionally, beta-glucans show promise in bone regeneration due to their ability to promote bone growth, inhibit osteoclastogenesis, enhance mesenchymal stem cell adhesion, and support osteoblast differentiation, thereby facilitating bone formation.^15^ While research on beta-glucans in dentistry is still limited, one study demonstrated that beta-glucans could reduce inflammation and alleviate alveolar bone loss in diabetic animals with periodontitis.^16^ These findings suggest that beta-glucan-containing wound dressings may represent promising therapeutic potential for improving wound healing in both medical and dental applications.

This study aimed to evaluate the effects of beta-glucan on pulpal wound healing by examining cell proliferation, cell migration, collagen synthesis, and mineralization in isolated human dental pulp cells.

## Materials and methods

### Isolation and culture of HDPCs

Human dental pulp cells (HDPCs) were obtained from healthy, non-carious third molars with no pulp disease, extracted for orthodontic purposes from patients aged 19–21 years (n = 3) following approval from the Human Experimental Committee, Faculty of Dentistry, Chiang Mai University, Thailand (No.7/2022). Prior to tooth extraction, patients performed an oral rinse with chlorhexidine mouthwash to minimize the microbial load in the oral cavity. Following extraction, the teeth were immediately rinsed with sterile saline. Strict aseptic protocols were adhered to throughout the tissue isolation process, including the preparation of guide grooves on the buccal aspects of the crowns of the extracted teeth using diamond burs (FG D8; Intensiv, Zurich, Switzerland). These grooves facilitated division of the teeth into two pieces using a chisel and mallet. The pulp tissue was then carefully collected using sterile instruments. Pulp tissues were digested with Collagenase I (Gibco, Gaithersburg, MD, USA) and Dispase II (Sigma–Aldrich, St Louis, MO, USA) for 45 min at 37 °C. Cells were cultured in complete alpha-minimum essential medium (Sigma–Aldrich) with 10 % fetal bovine serum (Sigma–Aldrich), 1 % penicillin-streptomycin (Sigma–Aldrich), and 100 mol/L l-ascorbic acid (Sigma–Aldrich) at 37 °C and 5 % CO2. Cells from the second to fourth passages were used. All experiments were performed in triplicate.

### Beta-glucan preparation

Beta-glucan from Euglena gracillis (Sigma–Aldrich) was used. A stock solution was prepared and stored at 4 °C using 0.1 % dimethyl sulfoxide (DMSO) (Sigma–Aldrich) as the vehicle. The stock solution was diluted with culture or differentiation medium, as required, and filter sterilized with 0.2-μm microfilters (Corning, Oneonta, NY, USA).

### Cell proliferation assay

HDPCs were seeded into 96-well plates at 5000 cells/well. After attachment, media was replaced with beta-glucan at 1, 2.5, 5, 7.5, and 10 mg/mL, with regular complete media as the negative control. A vehicle group examined DMSO's effect. To evaluate proliferation, 15 μL of alamarBlue® (Bio-Rad Laboratories, Hercules, CA, USA) were added to each well. Fluorescence was monitored at 24, 48, and 72 h using a plate reader (Tecan Trading AG, Männedorf, Switzerland) at 530 nm excitation and 590 nm emission. Percentage differences between control and treated groups were calculated. The three concentrations with the most pronounced proliferative effect were used in subsequent experiments.

After selecting the appropriate concentration of beta-glucan from the previous part, the following investigations were set into 5 experiment groups:

1. Control: HDPCs cultured in regular complete media
2. Vehicle: HDPCs cultured in regular complete media containing 0.1 % DMSO
3. BG 5: HDPCs cultured in regular complete media containing 5 mg/mL beta-glucan
4. BG 7.5: HDPCs cultured in regular complete media containing 7.5 mg/mL beta-glucan
5. BG 10: HDPCs cultured in regular complete media containing 10 mg/mL beta-glucan

### Wound healing assay

HDPCs were seeded in a 24-well plate at 30,000 cells/well. At 80 % confluence, a scratch was made using a sterile 100 μL pipette tip. Culture media with or without beta-glucan was added. The plates were incubated for 12 and 24 h under an automated live-cell imaging microscope (DMi8 microscope) (Leica Microsystems, Buffalo Grove, IL, USA) for live monitoring. Cell migration was assessed, and quantitative analysis was performed using Image J software.

### Investigation of integrin beta 1 expression using flow cytometry

To examine the role of integrin in cell migration, HDPCs were cultured in a 6-well plate at 300,000 cells/well. At 80 % confluence, scratches were made, and cells were treated with beta-glucan or complete media for 24 h. Cells were harvested, centrifuged, resuspended in FACs buffer, and incubated with FITC-conjugated integrin beta 1 antibody (Invitrogen, Carlsbad, CA, USA) for 30 min at 4 °C. Flow cytometry analysis was conducted using a CytoFLEX S flow cytometer (Beckman Coulter, Brea, CA, USA). Data were analyzed based on mean fluorescence intensity (MFI).

### Evaluation of mineralization production using alizarin red S staining

HDPCs were seeded in a 24-well plate at 20,000 cells/well. At 50 % confluence, scratches were made. Differentiation medium with beta-glucan (BG + diff) was used to stimulate mineralization for 14 days with medium changes every 3 days. Cells were fixed with 4 % paraformaldehyde and stained with pH 4.2 alizarin red S solution (Sigma–Aldrich). After incubation and washing, calcium deposits were measured by destaining with 10 % cetylpyridinium chloride monohydrate. The stained solution was measured using a spectrophotometer (Tecan Trading AG) at 550 nm.

### Gene expression using quantitative real-time PCR (qRT-PCR)

To evaluate DSPP, IL-10, and COL1, HDPCs at 200,000 cells/well were seeded in a 6-well plate and induced as previously described. Cells were collected on day 14. RNA extraction was performed with TRIzol (Invitrogen), followed by cDNA synthesis using the ReverTra Ace™ qPCR RT Kit (TOYOBO, Osaka, Japan). Gene expression was measured on the LightCycler® 480 II system (LifeScience, Roche, Indianapolis, IN, USA) using SYBR Green PCR master mix (SensiFAST™ SYBR® No-ROX Kit) (Bioline, Memphis, TN, USA). Relative expression levels were calculated by the 2^−ΔΔCT^ method, employing GAPDH as the internal control. Primer sequences are provided in Table 1.

**Table 1:** Primer sequences of genes used in the study. DSPP: dentin sialophosphoprotein. IL-10: interleukin 10. COL1: collagen type I. GAPDH: glyceraldehyde-3-phosphate dehydrogenase.

Gene	Primer sequences (5′-3′)
DSPP	Forward primer: TGG CGA TGC AGG TCA CAAT Reverse primer: CCA TTC CCA CTA GGA CTC CCA
IL-10	Forward primer: CCC AGA AAT CAA GGA GCA TT Reverse primer: CTC TTC ACC TGC TCC ACT GC
COL1	Forward primer: GAT GAT GCC AAT GTG GTT CGT G Reverse primer: CAG GCT CCG GTG TGA CTC GT
GAPDH	Forward primer: ACC ACA GTC CAT GCC ATC AC Reverse primer: TCC ACC ACC CTG TTG CTG TA

### Statistical analysis

One-way analysis of variance (ANOVA) was performed using SPSS Statistics 21.0 (IBM, Chicago, IL, USA), with Tukey's or Dunnett's T3 test applied for post-hoc analysis. Statistical significance was set at *P* < 0.05.

## Results

### Cell proliferation assay

**Figure 1:**
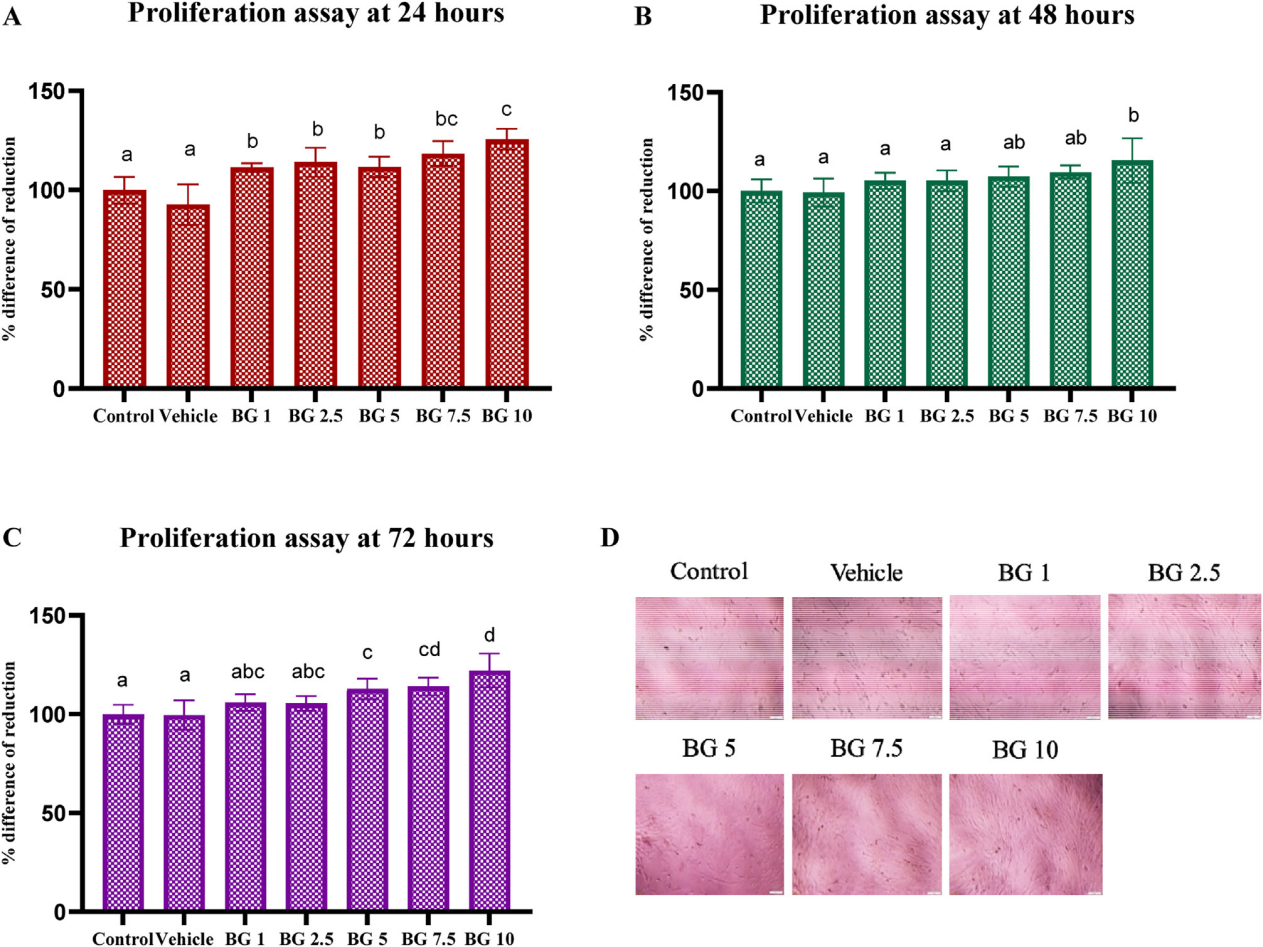
The proliferative effect of beta-glucan on HDPCs. (A) Cell proliferation at 24 h. (B) Cell proliferation at 48 h. (C) Cell proliferation at 72 h. (D) Cells at 72 h under an inverted-light microscope at 5× magnification Scale bar = 50 μm. Different letters in the graph represent significant differences between groups. BG: beta-glucan.

All concentrations (1, 2.5, 5, 7.5, and 10 mg/mL) of beta-glucan significantly increased cell proliferation relative to the control and vehicle groups at 24 h (*P* < 0.05) (Fig. 1A). At 48 h, cell proliferation trends increased in all beta-glucan groups, with the 10 mg/mL concentration showing significant stimulation compared to the control and vehicle groups (*P* < 0.05) (Fig. 1B). At 72 h, beta-glucan concentrations of 5, 7.5, and 10 mg/mL significantly increased cell proliferation relative to the control and vehicle groups (*P* < 0.05) (Fig. 1C). The 10 mg/mL concentration notably enhanced cell proliferation compared to the 1, 2.5, and 5 mg/mL concentrations (*P* < 0.05).

Based on the proliferation assay results, 5, 7.5, and 10 mg/mL of beta-glucan demonstrated the greatest cell proliferation. These concentrations were selected for the subsequent part of the experiment.

### Wound healing assay

**Figure 2:**
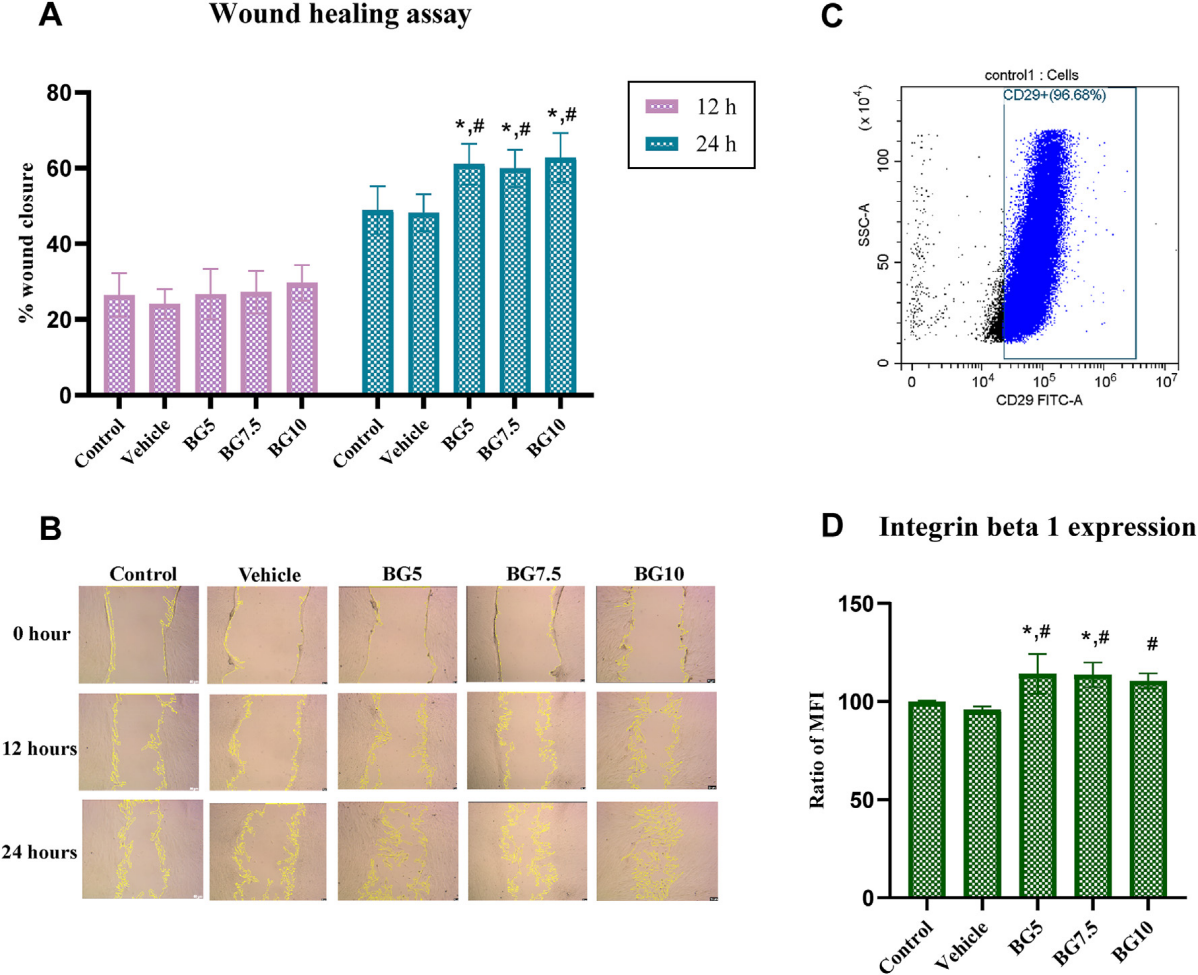
The migration potential of HDPCs after exposure to beta-glucan. (A) The migration potential results presented at the 12-h and 24-h time points. (B) Representative images of the migrated cells from the wound healing assay at 0, 12, and 24 h. The scale bar represents 50 μm. (C) The percentage of integrin beta 1 positive cells in HDPCs. (D) After 24-h exposure to beta-glucan, the expression of integrin beta 1 was assessed using MFI and presented as a percentage difference compared to the control. ∗ indicates significance compared to the control group while # indicates significance compared to the vehicle group. BG: beta-glucan. MFI: mean fluorescence intensity.

The results showed no significant difference was observed in wound closure between the groups at 24 h. However, at 24 h, the concentrations of 5, 7.5, and 10 mg/mL of beta-glucan significantly enhanced wound closure when compared to the control and vehicle groups (*P* < 0.05) (Fig. 2A). The 10 mg/mL concentration of beta-glucan showed the most extensive wound closure (*P* > 0.05). Similar repairing potentials were observed among the beta-glucan-treated groups.

### Integrin beta 1 expression

The findings indicated that beta-glucan at 5 and 7.5 mg/mL significantly increased the expression of integrin beta 1 when compared to the control group (*P* < 0.05). The concentrations of 5, 7.5, and 10 mg/mL of beta-glucan significantly induced the expression of integrin beta 1 when compared to the vehicle group (*P* < 0.05). Similar expressions were observed among the beta-glucan groups (Fig. 2D).

### Alizarin red S staining

**Figure 3:**
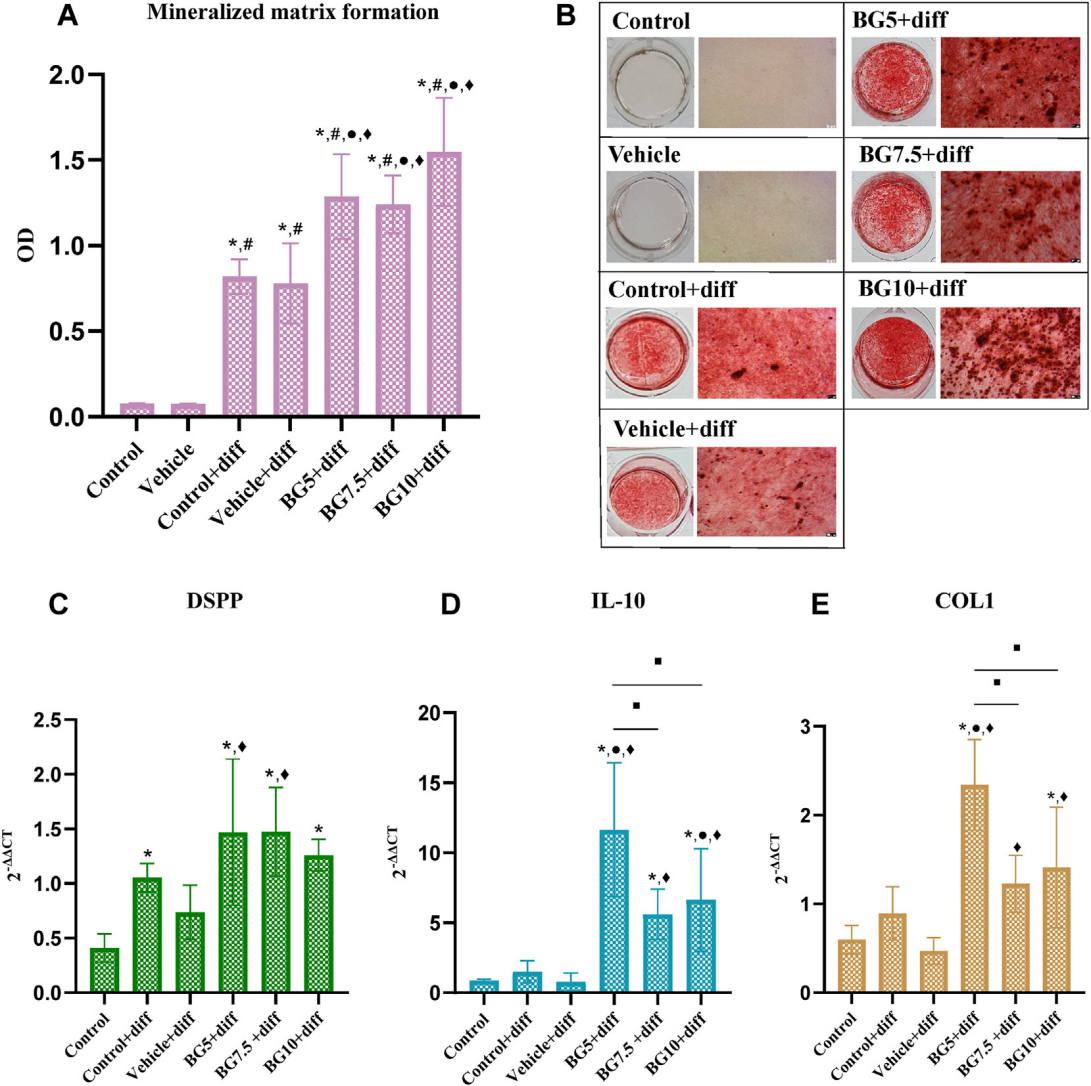
The effect of beta-glucan on mineralized matrix formation and collagen synthesis of HDPCs. (A) The quantitative analysis of mineralized matrix formation at 14 days. (B) Alizarin red staining of the experimental groups under a light microscope at a magnification of 5×. Scale bar = 50 μm. (C) The expression of the mineralization-related gene: DSPP (D) The expression of IL-10, and (E) The expression of collagen synthesis-related gene: COL1. ∗ indicates significance compared to the control group. # indicates significance compared to the vehicle group. ● indicates significance compared to the control + diff group. ♦ indicates significance compared to the vehicle + diff groups. ■ indicates significance between two different groups. OD: optical density. BG: beta-glucan. Diff: differentiation medium.

A significant increase in mineralized matrix formation was observed when compared to control and vehicle groups (*P* < 0.05). The addition of 5, 7.5, and 10 mg/mL of beta-glucan to differentiation media resulted in a significantly higher level of mineralized matrix formation when compared to control and vehicle groups under differentiating conditions (*P* < 0.05). Similar results were observed among the beta-glucan groups (Fig. 3A).

### The expression of mineralization-related and collagen synthesis-related genes

The expression of the DSPP gene significantly increased in the control, BG5, BG7.5, and BG10 groups under differentiating conditions compared to the control group (*P* < 0.05). Similar levels of DSPP expression were observed between the beta-glucan-treated groups and the control group under differentiating conditions. However, both the BG5 and BG7.5 groups showed a significant increase in DSPP expression compared to the vehicle group (*P* < 0.05) (Fig. 3C). Regarding IL-10 expression, all the BG5, BG7.5, and BG10 groups exhibited a significant upregulation of IL-10 expression compared to the control group (*P* < 0.05). Compared to the control group under differentiating conditions, the BG5 and BG10 groups displayed a significant elevation in IL-10 expression (*P* < 0.05). Additionally, all the BG5, BG7.5, and BG10 groups induced a significant increase in IL-10 expression compared to the vehicle group (*P* < 0.05) (Fig. 3D).

In terms of collagen gene expression, significant upregulation of COL1 was observed in the BG5 and BG10 groups under differentiating conditions compared to the control group (*P* < 0.05). When compared to the control with differentiation media group, only the BG5 group exhibited a significant increase in COL1 expression (*P* < 0.05). However, all the BG5, BG7.5, and BG10 groups displayed significant upregulation of COL1 when compared to the vehicle group in differentiation media (*P* < 0.05). There was also a significant difference in COL1 expression between the BG5 group and both the BG7.5 and BG10 groups (*P* < 0.05) (Fig. 3E).

## Discussion

The potential use of beta-glucan for pulpal wound healing was examined in isolated human dental pulp cells, focusing on cell proliferation, migration, collagen synthesis, and mineralization. Significant positive effects were observed at concentrations of 5–10 mg/mL, suggesting beta-glucan could promote healing in both soft tissue and mineralization, indicating its promise for regenerative endodontics.

Beta-glucans are complex polysaccharides found in various natural sources, known for numerous health benefits, including wound healing.^17^, ^18^, ^19^, ^20^ They mediate healing through several receptors, particularly dectin-1, found on immune and non-immune cells such as keratinocytes, fibroblasts, and dental pulp tissues.^19^^,^^21^^,^^22^ Despite extensive research on their wound-healing effects, no studies have specifically examined their impact on dental pulp cells until now.

Current dental treatments, especially VPT, emphasize regenerative trends where dental pulp cells are crucial. VPT aims to maintain pulp vitality and promote healing post-infection removal.^1^ Dental pulp cells initiate and coordinate healing responses, including proliferation, migration, differentiation into odontoblasts, and reparative dentin formation.^7^ Enhancing dental pulp cell potential is key to successful VPT outcomes.

In this study, beta-glucan enhanced dental pulp cell proliferation, migration, mineralization, and differentiation, with higher concentrations (5–10 mg/mL) being more effective. These findings are consistent with previous studies on beta-glucan's effects on other cell types. *In vitro* studies have shown that beta-glucan promotes cell proliferation across various cell types. For instance, 0.2 mg/mL mushroom-derived beta-glucan stimulated keratinocyte proliferation within 48–72 h.^23^ High concentrations (5 mg/mL) also showed greater efficacy in promoting fibroblast cell growth compared to lower doses.^24^

Cell migration is also crucial for wound healing, and previous studies have shown that beta-glucan enhances keratinocyte and fibroblast migration.^10^^,^^11^^,^^25^ Similarly, our study found that beta-glucan promoted HDPC migration in wound healing assays. Migration involves complex processes where integrins, acting as cell surface receptors, connect the extracellular matrix (ECM) to the cell's cytoskeleton and activate the FAK-Src pathway to facilitate movement.^26^, ^27^, ^28^ Previous research has associated increased cell migration with the expression of specific integrin subunits, including integrin alpha 3, alpha 5, and beta 1.^29^, ^30^, ^31^ In this study, the expression of integrin beta 1 correlated with improved HDPC migration following beta-glucan treatment, suggesting an association between these factors.

Collagen is essential for wound healing, providing structural support and maintaining tissue integrity within the ECM.^32^ IL-10 also plays a significant role in tissue formation and maturation, facilitating organized ECM deposition without compromising strength.^33^^,^^34^ Our study reveals that beta-glucan stimulates collagen synthesis gene (COL1) expression in HDPCs, aligning with previous research on human dermal fibroblasts.^35^ A consistent pattern of IL-10 and COL1 expression across beta-glucan-treated groups suggests IL-10's role in maintaining balance during wound healing.

For pulpal healing, a key goal is cell differentiation into mineral-producing cells. Various genes, including DSPP, DMP-1, and BMP, are involved in mineralization, with DSPP recognized as a marker of odontoblastic differentiation.^36^^,^^37^ Our study showed upregulation of DSPP expression and observed mineralization after beta-glucan treatment. While limited studies have examined beta-glucans' direct influence on mineralization, some reported mineralized matrix formation and bone deposition in beta-glucan-incorporated scaffolds.^15^^,^^38^^,^^39^ Further research is needed to explore beta-glucan's direct role in mineralization, including dentinogenic differentiation.

These finding suggest that beta-glucan may have a supportive role in processes relevant to dental pulp healing. The healing process following VPT involves complex cellular and molecular mechanisms that support pulp tissue regeneration and repair.^40^ Stimulating early cell proliferation, migration, and differentiation is critical for the success of VPT, and beta-glucan shows promising potential in these aspects. Its ability to enhance these processes highlights its possible application in improving the outcomes of VPT. However, further research is needed to fully understand the mechanisms behind beta-glucan's effects on HDPCs and to assess its long-term impact on pulp tissue regeneration and repair *in vivo*.

In conclusion, our study found that beta-glucan at concentrations of 5, 7.5, and 10 mg/mL demonstrated the ability to stimulate cell proliferation, cell migration, integrin beta 1 expression, and mineralization in HDPCs. The expression of IL-10, COL1, and DSPP genes was upregulated. This study highlights the potential of beta-glucan as a valuable enhancer of processes critical to dental pulp healing, paving the way for innovative approaches in regenerative endodontics.

## Declaration of competing interest

The authors have no conflicts of interest relevant to this article.
